# Cancer diagnostics: The journey from histomorphology to molecular profiling

**DOI:** 10.18632/oncotarget.11061

**Published:** 2016-08-04

**Authors:** Atif A. Ahmed, Malak Abedalthagafi

**Affiliations:** ^1^ Department of Pathology and Laboratory Medicine, Children's Mercy Hospital, Kansas City, Missouri, USA; ^2^ Brigham and Women's Hospital, Harvard Medical School, Boston, MA, USA; ^3^ The Saudi Human Genome Laboratory, Department of Pathology, King Fahad Medical City, King Abdulaziz City for Science and Technology, Riyadh, Saudi Arabia

**Keywords:** cancer diagnosis, histomorphology, targeted therapy, targeted molecular profile

## Abstract

Although histomorphology has made significant advances into the understanding of cancer etiology, classification and pathogenesis, it is sometimes complicated by morphologic ambiguities, and other shortcomings that necessitate the development of ancillary tests to complement its diagnostic value. A new approach to cancer patient management consists of targeting specific molecules or gene mutations in the cancer genome by inhibitory therapy. Molecular diagnostic tests and genomic profiling methods are increasingly being developed to identify tumor targeted molecular profile that is the basis of targeted therapy. Novel targeted therapy has revolutionized the treatment of gastrointestinal stromal tumor, renal cell carcinoma and other cancers that were previously difficult to treat with standard chemotherapy. In this review, we discuss the role of histomorphology in cancer diagnosis and management and the rising role of molecular profiling in targeted therapy. Molecular profiling in certain diagnostic and therapeutic difficulties may provide a practical and useful complement to histomorphology and opens new avenues for targeted therapy and alternative methods of cancer patient management.

## INTRODUCTION

The management of patients with cancer has always been a challenge to those working in the healthcare field. Despite significant research and practical advances that were made over the past few decades, the war against cancer is far from being over. Classic approaches to cancer treatment include a combination of surgery, radiotherapy, chemotherapy and hormonal therapy. With modern techniques and therapeutic options, the mortality and morbidity from cancer have decreased significantly in the past two decades. However, problems of therapy resistance and tumor progression and recurrence still plague many cancer survivors. The current treatment approach is based on a diagnosis of cancer that is rendered after pathologic analysis of the tumor and its characteristics. The process of histopathologic visualization of tumor cells, herein referred to as histomorphology, is the cornerstone of the pathologist labeling of a tumor as carcinoma, sarcoma or melanoma and is the basis of cancer treatment Advances in histopathology, which were largely fueled by curiosity regarding tumor morphology, have led to the development of an array of pathologic nomenclatures and classifications of different tumors.

In the past few decades, numerous research discoveries have been made in identifying genetic aberrations that have resulted in better understanding of the pathogenesis of malignancies and have allowed for more refined tumor diagnosis. The demonstration of specific genes mutations, fusion transcripts and chromosomal translocations has been of pivotal importance in the diagnosis of numerous cancers particularly sarcomas, some other solid tumors, lymphomas and leukemias. Genetic events in tumors result in the over-expression of signaling transduction pathways that regulate tumor proliferation, growth and spread. The link between genetic aberrancies and activation of signaling pathways and the mapping of different growth factors and their receptors have been elucidated in many tumors. These advances have allowed for more accurate diagnosis as well as exploration of novel therapeutics that target these genetic events and signaling pathways. The research on targeted therapy was sparked by discovery of imatinib mesylate and its broad anti-tumor effects [1, 2]. This tyrosine-inhibitory compound has been successful in targeting *KIT* mutations and related genetic aberrations in gastrointestinal stromal tumor (GIST), chronic myeloid leukemia and other c-kit expressing tumors. In many instances, the presence of *KIT* mutations in a tumor is equivalent to responsiveness to imatinib mesylate. Targeting *KIT* or other tyrosine kinase receptors has been at the epicenter of this exponential genomic research, and has opened new avenues in cancer classification and management.

## ROLE OF TUMOR HISTOMORPHOLOGY

Histomorphology is an ancient technique that has been essential in the identification and the diagnosis of all types of benign and malignant tumors. A portion of the patient's tumor tissue is fixed for several hours and subjected to histological processing technique that includes paraffin-embedding, microtome-sectioning and staining with hematoxylin and eosin stains that pathologists have been using for over a century [3, 4]. The stained sections are then microscopically reviewed by the pathologist who will draw upon his/her training and expertise to determine the nature and characteristics of the tumor. The basic triad of “histology, microscopy and the pathologist examination” results in a relatively short and inexpensive process. Currently, the cost of a single H&E slide averages $18 and most hospitals charge $10-45 per slide [5]. Financial expenses are mainly encountered in the initial laboratory set-up and training and salaries of pathologists and histotechnologists. The time period from obtaining a tumor specimen to the final pathologic diagnosis is 24 hours in urgent settings and is commonly 3 days in standard protocols.

**Figure 1:**
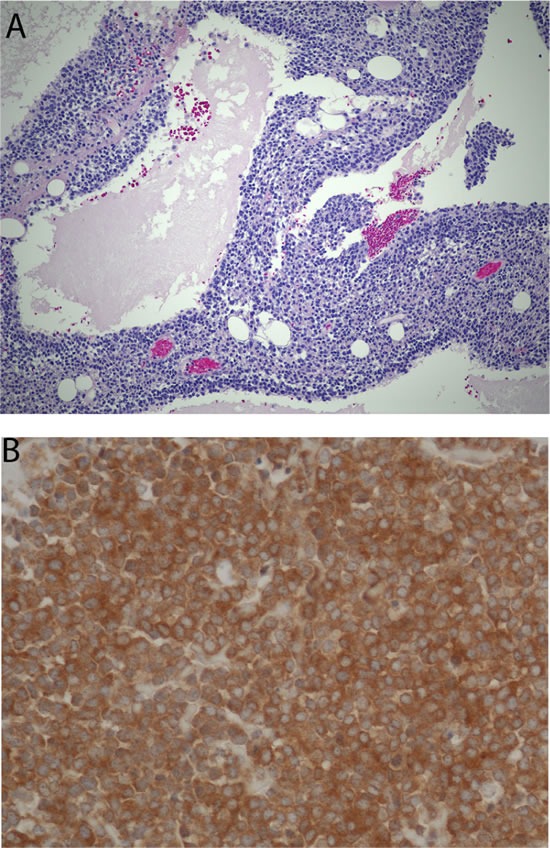
Histomorphologic appearance of a retroperitoneal tumor that could not be accurately classified by pathologists despite extensive work-up and opinions of multiple nationally-recognized experts (**A.** H&E, x100). The tumor responded to treatment with temsirolimus and an immunohistochemical stain revealed immunoreactivity with phosphorylated mTOR antibody test (**B.** x200).

Histomorphology has been valuable in the assessment of tumor topography and cellular morphology and in the classification and nomenclature of variety of disease entities. It also adds value to patients' management by identifying certain prognostic indicators such as lymphovascular invasion, infiltration of adjacent organs, necrosis, and mitotic rate. However, since the invention of light microscope in the sixteenth century and the discovery of hematoxylin and eosin stains more than a century ago, the process of histomorphology has remained as such without much change [3, 4, 6, 7]. Few but important advances in the field have been made, such as in the development of immunohistochemistry, *in situ* hybridization and digital pathology, all of which still rely on histomorphology and have added extra costs to patient's care [8, 9]. Furthermore, this classic approach to the cancer diagnostic management is sometimes unsatisfactory and has been criticized for various scientific and clinical reasons:

1. Cancer diagnosis often requires ancillary tests, such as immunohistochemistry and flow cytometry. These tests are performed in independent laboratory settings that incur additional costs. Immunostained slides and special stains typically cost the patient approximately $50 per single slide for in-house tests and an average of $200 per send-out test [5]. The number of slides processed in diagnostic biopsies, immunohistochemical studies, and subsequent resection specimens vary from few slides to more than 20 with an average of 10 slides per case, i.e. an average total cost of $2000 that also varies according to the cancer type [5]. Diagnostic immunohistochemical panels have been steadily increasing making it impractical for most hospitals to acquire all the inventory. These ancillary tests may delay the final diagnosis for additional few days, and the prolonged turnaround time in most hospital pathology laboratories has delayed treatment and resulted in unnecessary prolonged hospital bed stays.
2. There is no clear and direct scientific relationship existing between the tumor morphology and the response to therapy. The current classification of tumors vastly depends on histomorphology which has hampered any novel attempt to reclassify disease process or substantially develop new avenues in patient's management. Classifying malignancies into benign and malignant is sometimes ambiguated by the concepts of intermediate tumors, low grade malignancies, very low risk cancers and other tumors of variable clinical behaviors.
3. Some pathologists and treating physicians regard cancers as discrete morphologic entities whereas, in reality, cancer is a continuous disease process. Attesting to the latter fact, are the recent classifications of new diagnostic entities with intermediate morphological features that fall between two classic entities, such as ganglioneuroblastoma, B-cell lymphoma with features intermediate between diffuse large B-cell lymphoma and Burkitt lymphoma, B-cell lymphoma with features intermediate between diffuse large B-cell lymphoma and classical Hodgkin lymphoma, and transitional liver tumors that resemble hepatoblastoma and hepatocellular carcinoma [10–13]. Examples abound in the literature of tumors that have intermediate features or have a classic morphology with an “add-on” differentiation towards another morphology. Despite their apparently discrete morphology and immunophenotype, the fact that these tumors fall within a single pathway of carcinogenesis cannot be refuted.
4. Cancer histomorphology has become increasingly complex. Over the past few decades, the complexity of pathologic classification of cancers and the more vivid description of histomorphologic variants have substantially increased, fueled in part by the accumulation of rare consultation cases. Good examples include papillary thyroid carcinoma that has 12 variants, meningioma with 15 morphologic variants, and invasive breast carcinoma which has 20 described morphologic subtypes [14]. The subclassification of these variants is purely descriptive in most cases and does not affect the patient's prognosis or treatment. Furthermore, numerous tumors are encountered in daily practice whose nature has baffled pathologists and have since been labeled “undifferentiated sarcoma” or “undifferentiated carcinoma” (Figure 1).
5. Interpretation of histopathologic specimens can be subjective. A variable degree of discordance exists among pathologists in the interpretation of specimens depending on the specimen type and the expertise of the pathologist. A recent study published in the Journal of American Medical Association (JAMA) has reflected on the high degree of disagreement among breast pathologists and on the fact that their diagnosis may exhibit an inter-observer variability in up to 25% of cases [15]. The appreciation of the subjective nature of such interpretation and the alarming degree of pathologists' discordance have led to decreased clinician confidence, increased duration and complexity of pathologists training and eventually to the increased use of diagnostic tests for which the patient will ultimately bear the costs.

## IMPACT OF MOLECULAR TESTING

Molecular cancer studies have expanded beyond understanding tumor pathogenesis to the clinical laboratory setting where tests are occasionally requested to assist in the diagnosis of various tumors and aid in the patient's management. The identification of specific genetic events in tumors has fueled the development and rapid growth of molecular diagnostic tests that are based on polymerase chain reactions, immunohistochemistry or other simple techniques. The identification of *EWS/FLI1* chromosomal translocation and *KIT/PDGFR* mutations are indispensable in confirming the diagnosis of Ewing's sarcoma and GIST respectively. Furthermore, molecular tests often provide valuable information about the patient's prognosis and expected survival outcome. Combination of molecular and immunohistochemical tests has resulted in the molecular subclassification of medulloblastoma and breast cancer [16]. The identification of *FLT3* mutations in acute myeloid leukemia, exon 11 mutations in GIST tumors and alpha thalassemia x-linked protein (ATRX) and isocitrate dehydrogenase expression in brain gliomas is clinically useful to identify aggressive cancers of different prognosis and help guide appropriate chemotherapy [17–19]. These diagnostic tests are designed to complement rather than replace routine tumor histomorphology and have proven their diagnostic value in most hospitals. However, expensive molecular tests and detailed immunohistochemical panels are not widely available, thus reflecting an economic disadvantage to their use in small hospitals [20]. Notwithstanding, molecular diagnostic tests are increasingly being developed and utilized as diagnostic as well as therapeutic tests.

## RECLASSIFICATION OF TUMORS

The expansive research into cancer genetic background has led to the identification of shared genetic events in tumors with diverse histologic appearances. These findings have prompted researchers and pathologists to consider classifying these tumors according to their “genomic signature” or “genetic profile”. A good example is the identification of *EWS* gene rearrangements in Ewing's sarcoma, desmoplastic round cell sarcoma, extraskeletal myxoid chrondrosarcoma, myxoid liposarcoma, clear cell sarcoma and several other cancers [21]Similarly, *KIT* mutations and KIT protein expression has been identified in numerous tumors including GIST and other tumors unrelated to GIST [22]. Such tumors become amenable to inhibition by imatinib mesylate and other tyrosine kinase inhibitors. *SMAR CB1 (INI-1)* deletions are increasingly being identified in multiple tumors [23]. Thus, the grouping of these tumors according to their shared characteristic genetic events is a justifiable approach in attempting to understand their molecular carcinogenesis and has prompted alternate classification of tumors based on their genetic profile, e.g. *ALK*-rearranged, *KIT*-mutated, *EWS*-rearranged, and *INI*-*1* deleted. Similarly, the identification of translocations and shared gene signatures has provided for the reclassification of rhabdomyosarcomas (RMS) into fusion-positive RMS and fusion negative-RMS, which includes embryonal RMS and fusion negative alveolar RMS [24]. Table 1 demonstrates a novel method of classifying cancers according to the characteristic genetic mutations and signaling profiles they express.

**Table 1:** Classification of common cancers based on their molecular profile

A: Examples of characteristic genetic and cytogenetic events:	
Kit Mutations	GIST, Seminoma, Adult mastocytoma, Acute myeloid leukemia, Sinonasal NK cell lymphoma
**BRAF mutations**	Thyroid cancer, Melanoma, Colorectal carcinoma, Hairy cell leukemia, Brain gliomas
**ALK rearrangements**	Anaplastic T-cell lymphoma, Diffuse large B-cell lymphoma, Lung adenocarcinoma, Familial neuroblastoma, Inflammatory myofibroblastic tumor, Epithelioid inflammatory myofibroblastic tumor, Spitz tumor, systemic histiocytosis, Renal cell carcinoma
**EWS rearrangements**	Ewing's sarcoma family, Desmoplastic round cell sarcoma, Extraskeletal myxoid chondrosarcoma, Myoepithelial tumors, Clear cell sarcoma, Angiomatoid fibrous histiocytoma, Myxoid liposarcoma
**BAF-deficient or dysregulated tumors**	Malignant renal and extrarenal rhabdoid tumors, Atypical rhabdoid teratoid tumor, Epithelioid sarcoma, Epithelioid malignant peripheral sheath tumor, Schwannoma, Renal medullary carcinoma, Sinonasal carcinoma, Vulvar carcinoma, Thoracic carcinoma, Small cell carcinoma of the ovary hypercalcemic type, Synovial carcinoma, Endometrial dedifferentiated carcinoma, Poorly differentiated lung carcinoma
**ATRX mutations**	Brain gliomas, neuroendocrine tumors, Osteosarcoma, Liver angiosarcoma, Leiomyosarcoma

B: Examples of common signaling transduction pathways over-expressed in various cancers:	
**EGFR**	Lung carcinoma, Breast carcinoma, Head and neck carcinoma, Colon cancer
**IGFR**	Breast cancer, Ewing's sarcoma, Rhabdomyosarcoma, Fibrosarcoma, GIST
**mTOR**	Renal cell carcinoma, Breast carcinoma, Sarcomas, Medulloblastoma, Glioblastoma
**MAP/PI3k/Akt**	Breast carcinoma, Endometrial cancer, Sarcomas
**VEGF**	Lung carcinoma, Colorectal carcinoma, Gynecologic cervical, Ovarian or fallopian tube carcinomas
**Wnt**	Colorectal carcinoma, Desmoids, Breast carcinoma, Brain tumors
**Sonic Hedgehog**	Pancreatic carcinoma, Basal cell carcinoma, Colorectal carcinoma, Metastatic prostate carcinoma, Medulloblastoma
**Hippo**	Osteosarcoma, Rhabdomyosarcoma, Liver cancer, Medulloblastoma
**PDL1**	Non-small lung cancer, Breast cancer, Hodgkin's lymphoma, B-cell lymphomas, Renal cell carcinoma, Gastrointestinal malignancies

An interesting observation is that some genetic mutations are identified among tumors of various biologic behaviors including benign tumors, low grade and high grade malignancies. *KIT* mutations occur in benign entities such as mastocytosis as well as malignant tumors such as GIST. These tumors are treated differently regardless of the underlying genetic events. In these instances, identifying molecular events, without histomorphology, cannot predict the tumor's biologic behavior.

## TARGETED THERAPY

Targeted therapy is based on the inhibition of up-regulated mutational events and signaling pathways in cancers. Numerous cell growth factors and their receptors converge on the PI3K/Akt/mTor pathway and other intracellular pathways, which are widely activated in numerous cancers [25]. Preclinical and clinical studies have focused on targeting these mutated genes, growth factor receptors and intracellular signal transduction pathways over-expressed by the tumor cells [26]. Good examples include the use of mTor inhibitors in renal cell carcinomas, epidermal growth factor receptor (EGFR) and PDL1 inhibitors in non-small cell lung carcinoma and the experimental use of insulin growth factor-1 receptor (IGF1R) inhibitors in sarcomas [26].

In most cases, targeted therapy is offered to patients after the failure of routine or standard chemotherapy. Clinical trials are commonly conducted on patients previously heavily treated by conventional chemotherapy or on patients whose tumors have progressed despite adequate chemotherapy. In few instances, initial treatment with targeted therapy is offered for tumors which are considered to be resistant to conventional chemotherapy. In this regard, targeted therapy has revolutionized the treatment of renal cell carcinoma and GIST [27, 28]. The fact that many cancers share similar molecular pathways and genetic profiles makes them amenable to the same type of targeted therapy. Clinical trials of PI3K/mTor inhibitors have been conducted in variety of cancers including breast cancer, renal cell carcinomas, and hematological malignancies. Receptor tyrosine kinase inhibitors, such as imatinib, sunitinib, and dasatinib, are used to treat GIST, acute myeloid leukaemia (AML), systemic mastocytosis, meningioma, dermatofibrosarcoma protuberans, and melanoma [29].

## PREDICTIVE TESTS FOR PERSONALIZED TARGETED THERAPY

Not all tumors respond to the same targeted therapy and thus predictive tests are needed to identify patients and tumors that are most likely to respond to that particular inhibitory therapy [30]. This selective step has become increasingly important with the results of clinical trials being less satisfactory than expected. Laboratory diagnostic tests to identify targetable biomarkers are currently limited but more are being developed. Few notable successes in this field include the identification of trastuzumab-responsive breast cancers by testing for Her2-neu, *BCR/ABl* translocation in chronic myeloid leukemia, and *KIT* mutations in imatinib-responsive GIST [31]. Additional molecularly targetable biomarker discoveries include *BRAF* mutation testing for variable tumors like melanoma and craniopharyngioma and anaplastic lymphoma kinase (ALK) inhibitors and epidermal growth factor receptors in various subtypes of lung cancer [32, 33].

**Figure 2:**
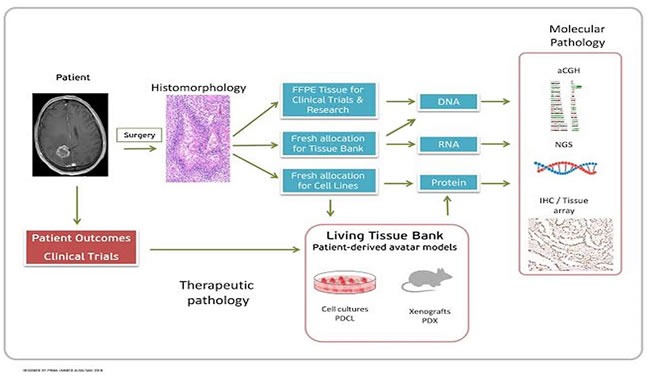
Triage of clinical specimens to different laboratories in the integrated approach to patient-oriented molecular and histomorphologic diagnosis for targeted real time management

Numerous other tumors are lagging behind in the discovery and standardization of similar predictive tests. Immunohistochemical tests provide some promise as predictive tests (Figure 1) but lack the standardization and inter-observer agreements that often hurdle the development in this area [34]. King et al have summarized several ways of identifying high expression of IGF1R in tumors as predictive tests [35]. Other attempts have been made in identifying renal cell carcinomas that are responsive to mTor inhibitors and sarcomas that are responsive to IGF1R inhibitors [36, 37]. Recent advances in high throughput tumor genomic sequencing have enabled more accurate mapping of molecular phenotypes and offered potential usefulness as predictive tests for tumor responsiveness to targeted therapy [38]. The use of mouse avatar models and testing of chemotherapeutic agents in cell cultures obtained from patient's cancer has recently emerged as a novel option for selecting the most appropriate treatment plan and thus avoiding unnecessary toxicity [39]. A new role of the pathologist, particularly in pediatric hospitals, is to triage precious fresh cancer specimens in different media for variety of diagnostic tests and therapeutic uses (Figure 2).

## MOLECULAR PROFILING OF TUMORS

Biologic pathways and genetic events in cancers are multiple and reveal complex interactions that affect tumor pathogenesis at different levels including initial tumorigenesis, sustained proliferation, inhibition of apoptosis, invasion and metastasis. Each tumor is expected to over-express specific genetic components or biologic pathways that are unique to the tumor, i.e. “molecular profile”. However, standard genomic or molecular profiling yields massive amount of data that are non-essential and difficult to decipher and less than 20% of such data can be described as clinically relevant [40]. Only a portion of the molecular profile can be inhibited by targeted therapy and can hence be labeled as the targeted or therapeutic molecular profile (TMP). The TMP components include signal transduction pathway members, hormones, angiogenesis and apoptosis pathway members and immune system modulators. Specific genomic profiles, gene expression modulators, fusion proteins or gene mutation signatures of a tumor can be included in this profile as long as they are “actionable” or “targetable”. Utilizing powerful computational methods, several web-based databases are becoming available that categorize all known protein targets including potential targets and approved drugs [41, 42]. The power of next-generation DNA sequencing methods in identifying targetable gene mutations has recently been appreciated in several studies that attempted to identify all the actionable mutations in well known cancers [43–47]. Thus, with modern technological advances and computerized software, the concept of TMP may be progressively realized and practically analyzed as more opportunities for targeted therapy are developed.

Molecular profiling has been used in large clinical centers for the targeted therapy treatment of tumors that are resistant to standard chemotherapy, progressed or metastasized despite adequate chemotherapy and in patients who do not tolerate standard chemotherapy [48]. In such instances, profiling of the metastatic tumor may be more important than the initial diagnostic specimens [49]. Complete tumor profiling at multiple platforms (e.g. gene sequencing, gene copy number and protein expression) may be performed in order to identify the proper therapeutic targets.

Molecular profiling can also offer insight into tumor classification. For example, osteosarcoma and Ewing's sarcoma and a variety of other sarcomas over-express IGF1R. Hence, these tumors can grouped together and treated with IGF1R inhibitors [50, 51]. The molecular profile or genetic signature may also be used to identify the tumor or cancer type. This concept has been recently tested by some investigators who were able to transform genomic expression data into disease diagnostic categories with 95% accuracy, a process that may alternatively be named “reverse profiling” [52]. However, until this potential is fully achieved, molecular profiling, in its current status, does not distinguish benign *versus* malignant tumors with high accuracy in all cases but can significantly complement tumor morphology in the overall diagnostic and therapeutic assessment of cancer.

## NEW WINDOW INTO CANCER PATIENT MANAGEMENT

Resistance to treatment is a common phenomenon in treating patients with cancer and can develop de novo or during the course of treatment. The development of targeted therapy has also been confounded by resistance that extended to the use of second- or third-generation molecular therapies. Although resistance to targeted therapy may be due to several pharmacologic and molecular aberrations within the tumor cells, the delay in starting such treatment until failure of previous chemotherapy may account for some of the de novo resistance mechanisms. However, an option exists for initiating targeted therapy based solely on the tumor's TMP and without prior chemotherapy. Targeted therapy is frequently considered as the last reserve in the fight against cancer and has seldom ever been tested as an initial treatment choice. Initiating treatment with targeted therapy in lieu of standard chemotherapy has been accomplished in the treatment of gastrointestinal stromal tumors, renal cell carcinomas and few other tumors that are known to be natively resistant to standard chemotherapy [27, 28]. Following this trend, other cancers may also be treated with targeted therapy regardless of chemotherapy options.

The use of imaging technologies such as Computed Tomography and Positron Emitted Tomography has already influenced early cancer detection and management [53]. Future advances in the field of molecular imaging, integrated diagnostics, biology-driven interventional radiology and theranostics in combination with molecular profiling and targeted therapy may open a new window into patient's management potentially bypassing the need for detailed histopathologic cancer identification and standard chemotherapy [53]. In this process cancer is diagnosed through the analysis of patient symptomatology and imaging studies. The tumor is then biopsied or surgically resected. The tumor specimen is subjected to detailed molecular profiling and the most appropriate targeted therapy is offered based on the results of the tumor's TMP. Few clinical trials have addressed the possibility of targeted therapy as first-line treatment option. As a first-line treatment for advanced EGFR mutation-positive non-small cell lung carcinoma, Erlotinib was found to be superior to standard chemotherapy and conferred a better progression-free survival in patients [54]. Thus, by obviating the need for standard chemotherapy, molecular profiling and targeted therapy may provide treatment options with limited histomorphology.

## BENEFITS AND PITFALLS OF MOLECULAR PROFILING

The use of molecular diagnostic tests provides a strong argument for evidence-based practice of medicine and yields a logical correlation between the molecular profile and response to targeted therapy. In combination with histomorphology, these methods can significantly complement cancer diagnosis. Non-histologic molecular diagnostic tests can be performed on the patient's tumor tissue directly or on cells harvested from body fluids and include techniques based on DNA/RNA, protein assay methods, and biologic assay methods (Figure 2). Next generation sequencing methods are leading cancer profiling research [55, 56], and a few advances in non-morphologic proteomic methods have also been made [57–59]. In addition to their value in yielding diagnostic and prognostic information, molecular profiling and gene expression signatures have also been successfully used to differentiate between benign and malignant breast and thyroid lesions with high accuracy [60, 61].

In cancer management, the toxicity and related healthcare costs associated with the use of personalized targeted therapy are less pronounced than those of standard chemotherapy. Serious toxicities have been rarely reported, that generally reflected poor patient selection due to lack of reliable predictive tests. The selective use of cancer agents directed against a specific molecular target on cancer cells has been reported to be associated with a lower incidence of toxicity and lower healthcare costs compared with the use of less-specific targeted agents, including general angiogenesis inhibitors and chemotherapeutic agents [62]. Thus, the success of targeted therapy depends on appropriate patient selection which itself relies on the improved development of personalized predictive diagnostic tests [63]. Further development of diagnostic tests is feasible with the collaborative effort of drug manufacturers and diagnostic companies that help maintain a cost-effective approach to cancer management [12].

Conceivably, the use of molecular diagnostic tests is currently associated with the requirement for large amounts of tissue that are needed for different techniques, including precious fresh or frozen tumor tissue [64]. In contrast, cancer histomorphology can reliably be performed on small tissue samples. The ability to extract DNA from formalin-fixed paraffin-embedded tissue has encouraged many advances in the field. However, such DNA samples, particularly when used in next generation or whole exome sequencing methods, is considered to be of inferior quality and often leads to artificial sequence alterations [65, 66]. Furthermore, cancer cells exhibit genetic heterogeneity in the sense that different cancer cells from the same tumor may have different mutation types according to their maturity and genetic evolution. Such genetic variation across individual tumors, intratumoralheterogeneity, has important implication for cancer progression and has been detected at the level of circulating tumor cells [67–69]. Although the drawbacks currently limit the utilization of molecular testing, future advances may overcome these difficulties. Promising results have been reported from the use of fine needle cytology specimens and from the detection of circulating tumor cells highlighting a stronger role of molecular testing in these areas [70, 71].

The high cost and the lengthy turnaround time have thus far limited the widespread use of next generation sequencing platforms and their clinical applications. The price cost of DNA sequencing and microarray tests is currently fairly high [72], and varies according to the instrument and platform type. In contrast, cancer histomorphology may provide more useful information at the fraction of the cost. Furthermore, molecular sequencing is prone to errors that arise during sequencing or interpretation [73]. Comprehensive analysis requires the services of an experienced bioinformatics professional, which further increase testing costs. However through advances in targeted therapy and in certain diagnostic and therapeutic difficulties, targeted tumor profiling may ultimately result in overall direct and indirect benefits for the patients and the healthcare system and may provide considerable time and cost savings in the overall management of cancer patients. Technologic improvements in the future may eventually lead to cost decrease and shorter turnaround time. Recently, a rapid 26-hour whole genome sequencing method, STATseq, has enabled, in combination with the clinical phenotype, a complete molecular diagnosis of inherited genetic diseases with more than 99% sensitivity [74, 75]. A similar method may be implemented in cancer diagnosis that allows for screening of fewer selected genes or a smaller TMP panel in combination with histomorphology with the potential for faster results.

## THERAPEUTIC PATHOLOGY AND INDICATIONS OF MOLECULAR PROFILING

Given the drawbacks in cancer histomorphology and molecular profiling, there are currently no perfect methods to guide cancer treatment. However, the field of therapeutic pathology and its role in the treatment of cancer is continually evolving. Cancer histomorphology will likely continue to be the basis of cancer diagnosis and management and reconcile with the growing trend in molecular diagnosis and targeted therapy by offering a combined or integrated tumor classification scheme. A more affordable approach would be to limit molecular testing to the identification of certain therapeutic and prognostic biomarkers. In this scheme, a basic morphologic cancer diagnosis is followed by ancillary immunohistochemical or other tests that may offer prognostic or therapeutic information (Figure 2). The College of American Pathology has recently established protocols for reporting certain biomarkers in different cancers [76]. This approach can also help in further classification of certain malignancies as recently highlighted in the new World Health Organization classification of hematopoietic malignancies which has categorized new entities with integrated nomenclature such as “myeloid neoplasm with *PDGFRB* rearrangement”, B-cell lymphoma with *IRF4* rearrangement and high grade B-cell lymphoma with *MYC* and *BCL2* and/or *BCL6* rearrangement [77]. While this integrated scheme offers a more comprehensive cancer assessment, it further increases the cost of cancer diagnosis and management.

Comprehensive molecular profiling based on next generation sequencing methods is currently routinely not practical but may be helpful in certain clinical situations:

1. To detect targetable mutations in otherwise difficult to treat, chemoresistant or metastatic tumors. In these cases, identifying tumor TMP from metastatic specimens is more important than tumor profiling from initial diagnostic specimens [49]. The practical use of mTOR inhibitors has been discussed in several clinical studies of metastatic renal cell carcinoma and ependymoma expressing the mutated gene or abnormal protein [78, 79].
2. To provide targetable options for tumors than could not be histologically adequately classified. In these situations, targeted therapy may be offered as an initial treatment option. This is particularly helpful in pediatric cancers where the robust genomic sequencing methods have helped solve the diagnostic odyssey and alleviate parents' anxiety [80–82]. Several ambitious projects in pediatric oncology programs across the USA, such as BASIC3 of Texas Children's Hospital, have suggested that clinical applications of cancer genomic testing have grown beyond the infancy stages [83–85].
3. To determine the origin of a metastatic tumor of unknown primary [86, 87]. Gene expression profile and transcriptome signature analysis were able to identify the site of origin of metastatic tumors with more than 95% accuracy [88, 89].
4. To provide a refined diagnosis in cases of undifferentiated malignancies. In a recent transcriptome analysis of 19 undifferentiated sarcoma of the thorax, identifying SMARCA4 mutations has placed these tumors along with malignant rhabdoid tumors rather than lung carcinomas [90].
5. To detect circulating genetic material or tumor cells in liquid biopsies from patients with metastatic disease [91];

With future advances in test costs and turnaround time, tumor profiling methods may become more affordable and practical and offer more opportunities in therapeutic pathology. There are at the present not enough data to show the life cost benefit of pure molecular profiling *versus* histomorphology in the management of cancer patients. However, with wider availability of molecular tests and development of successful targeted therapy, the role of cancer histomorphology may gradually decrease giving way to laboratory-based molecular tests that may be interpreted by molecular pathologists and non-MD scientific personnel.

## CONCLUSIONS

The development and applications of cancer profiling methods will be the cornerstone of targeted cancer research offering novel methods of cancer classification and treatment. Comprehensive or limited cancer profiling offers an alternative approach to treat patients with difficult cancers and can supplement or supersede classical management methods such as histomorphology and chemotherapy. The success of this approach will be further appreciated through the growing research in targeted therapy and the development of accurate and less expensive molecular diagnostic tests that accurately predict the response to targeted therapy. Given the rapid pace in which advances in tumor diagnostics are expanding, pathologists, researchers and clinicians should be willing to consider novel ways of approaching cancer treatment.

## Abbreviations

ABL: Abelson murine leukemia oncogene homolog 1
AKT: protein kinase B
ALK: Anaplastic lymphoma kinase
ATRX: alpha thalassemia x-linked protein
BAF: Brg/Brahma-associated factors
BCR: Breakpoint cluster region
BRAF: Murine sarcoma oncogene homolog B
EGFR: Epidermal growth factor receptor
EWS: Ewing's sarcoma oncogene
FLI1: Friend leukemia integration 1 transcription factor
FLT3: fms-like tyrosine kinase 3
H&E: Hematoxylin and eosin
Hippo: Hippopotamus-like phenotype
GIST: Gastrointestinal stromal tumor
IGFR: Insulin growth factor receptor
INI1: Integrase interactor 1
KIT: CD117 kinase receptor
mTor: Mammalian target of rapamycin
PDGFR: Platelet-derived growth factor receptor
PDL-1: Programmed death ligand-1
PI3K: Phosphatidylinositol-3 kinase
RTK: Receptor tyrosine kinase
SMAR CB1: SWI/SNF-related matrix-associated actin-dependent regulator of chromatin subfamily B member 1
VEGF: Vascular endothelial growth factor
WNT: Wingless-type integration site
